# BTEX detection with composites of ethylenevinyl acetate and nanostructured carbon

**DOI:** 10.3762/bjnano.8.100

**Published:** 2017-05-04

**Authors:** Santa Stepina, Astrida Berzina, Gita Sakale, Maris Knite

**Affiliations:** 1 Institute of Technical Physics, Faculty of Material Science and Applied Chemistry, Riga Technical University, Paula Valdena Street 3/7, Riga, LV-1048, Latvia

**Keywords:** benzene detection, polymer–nanostructured carbon composites, volatile organic compound (VOC) sensors

## Abstract

By using a solvent-based method composites of ethylenevinyl acetate copolymer and carbon black (EVA–CB) were synthesized for sensing BTEX (benzene, toluene, ethylbenzene and xylene) vapours. The composites were characterized using atomic force microscopy (AFM) in an electroconductive mode. Gas sensing results show that EVA-CB can reproducibly detect BTEX and that the response increases linearly with vapour concentration. Compared to gas-sensing measurements of gasoline vapours, the responses with toluene and ethylbenzene are different and can be explained by varying side chains of the benzene ring.

## Introduction

Solvents such as toluene, benzene, as well as various fuels are widely used in factories and in manufacturing. Many of these compounds are hazardous to humans [1–3] and have a significant impact on the environment, i.e., causing damage to the ozone layer [4–6]. Because of the hazardous nature of these solutions, in the United States the Occupational Safety and Health Administration (OSHA) has determined permissible exposure limits (PEL) for different substances. For benzene the 8 hour workday PEL is 1 ppm while for a 15 minute exposure it is 5 ppm [7]. These are quite low concentrations that humans may not be able to sense.

Previous [8–9] measurements of various fuels, such as diesel fuel and petrol, with composites of ethylenevinyl acetate copolymer and carbon black (EVA–CB) were carried out in order to investigate the response of EVA–CB and the ability to distinguish between different types of fuel. Obtained results demonstrated a clear difference between diesel fuel and gasoline. The different gasoline content was further investigated and results showed that gasoline contains more than 60% of benzene derivatives (BTEX and others). Due to the potentially harmful health effects of benzene and its derivatives, their content in gasoline is regulated. However, there is still no ideal gasoline quality detector. Similarly, in air pollution monitoring there are still problems with fast detection and precise determination of the concentration of volatile organic compounds (VOC). Therefore, with the wide usage of VOC, especially BTEX, there is a strong need for the development of new sensors that could easily, precisely and quickly determine VOC and their concentration.

One of the examples for a VOC sensor is a polymer-based nanostructured composite filled with electroconductive nanoparticles. Compared to gas sensors based on metal oxides this type of sensor ensures a much easier usage because polymer-based composites do not require high operating temperatures and work at room temperature. The gas-sensing measurement depends on the polarity of the polymer and is different for polar and non-polar solvents.

Sensors for VOC and various gases have been widely researched around the world [10–18]. Mondal et al. [19] in their study about VOC sensing materials used hybrid composites. The use of nanocarbons increases the detection range as well as the electrical conductivity of the chemiresistors and decreases the temperature dependence. Hybrid composites were made of poly(dimethyl siloxane) (PDMS) with nanocarbon black (NCB) and carbon nanotubes (CNT) as fillers and these composites showed reversible gas-sensing measurements on BTEX. They also point out that the increased electrical conductivity, decreased temperature dependence of conductivity, and stretchability will be useful in stretchable electronics.

In our previous works [8–9,20–23], we determined that the EVA–CB sensing measurement increases when the layer thickness of EVA–CB is decreased or the vinyl acetate (VA) content in the copolymer is increased.

In addition, the environmental conditions can also influence the sensing effect. In particular, it was determined that relative humidity affects the sensing measurements [21]. In near 100% relative humidity proton conductivity dominates over the tunnelling current conduction mechanism typical for polymer–carbon nano-filler composites. This proves that sensing measurements do not increase with increased humidity, but the full mechanism is still unknown.

The aim of this study is to create a sensing material that could be used for BTEX sensing at room temperature and evaluate the EVA–CB capability in sensor applications in fuel quality control. In this study, a conductive structure of EVA–CB composites is characterised using AFM electroconductive measurements in order to determine dispersion, aggregate size and distribution of carbon nanoparticles. Gas sensing measurements are made using different concentrations of BTEX vapours as well as of gasoline vapours.

## Experimental

### Materials and composite structure

**Figure 1:**
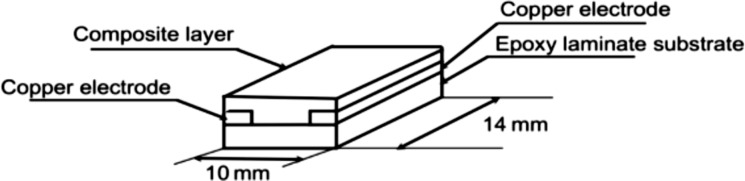
Schematic structure and dimensions of an EVA–CB sample. Reproduced with permission from [20], copyright 2011 Versita Warsaw and Springer-Verlag Wien.

The sensor material used is a nanostructured composite of a polymer matrix filled with electroconductive particles. The matrix material is ethylenevinyl acetate (EVA) copolymer and the conductive nanoparticles are carbon black. EVA (Sigma–Aldrich) with a vinyl acetate content of 40% was used as the polymer matrix for the nanostructured composite. EVA was chosen because of its dual polarity. EVA has a polar part (vinyl acetate) and a non-polar part (ethylene). This allows one to detect polar as well as non-polar VOC vapours. Carbon nanoparticles (CB; PRINTEX XE-2) with average particle size of 30 nm were used as electroconductive filler. The composite EVA–CB was synthesised via the solution method using 50 mL of chloroform. The mixture was made with 4 g EVA and 0.31 g of CB, which corresponds to 7.75 phr (parts per hundred rubber). The EVA–CB solution was applied through dip coating on an epoxy laminate substrate with two copper electrodes (Figure 1). The samples were dried at room temperature [20]. The schematic structure and the dimensions are illustrated in Figure 1.

### Methods

Gas-sensing measurements were carried out using a Kin-Tek FlexStream automated permeation tube system with a benzene permeation tube and synthetic air as carrier gas. A specialized sample holder and an Agilent 34972A (Keysight 34972A data acquisition/data logger switch unit) were used to measure the electrical resistance of the sample during the tests. The Agilent 34972A also was used for gasoline and ethylbenzene vapour measurements. The composite was characterized using the electroconductive mode of an atomic force microscope (AFM; NT-MDT, Smena) and the degree of dispersion of the conductive particles was indirectly determined by a modified method from [24]. The conductive mode of AFM shows us the electroconductive channel system of the sample, which indirectly quantitatively characterizes the degree of dispersion. The investigated area size is 100 × 100 μm, the feedback system gain is 1.0, the probe movement speed is 142 μm/s, the image resolution is 512 pt, the applied voltage is 0.5 V and the set point is +2.

From [24] the composite is characterized by acquiring a microscope image and dividing it into 12 squares, which are further mathematically processed so that the indices for particle distribution (dIndex), for particle agglomeration (sIndex), and for an overall sample characterization (compIndex) can be calculated. We have adapted the formulas to the images acquired in the electroconductive mode of AFM. The main difference is that with AFM we get a square image and we divide it into 3 × 3 squares. Also AFM provides a more detailed image compared to a simple optical microscope, so the threshold for agglomerates was decreased to five pixels (approximately 980 nm). The resulting formula for dIndex is:

[1]
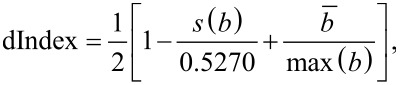


where *b* is the number of electroconductive channels observed for each of the nine sections of the image, expressed as a percentage. *s*(*b*) is the standard deviation of *b*, and 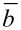 is the arithmetic mean of *b*. The value of max(*b*) is the maximum concentration of electroconductive channels in any image section. The constant 0.5270 is the largest possible standard deviation for nine numbers that range between 0 and 1, namely the set of five zeros and four ones.

For sIndex the formula is:

[2]
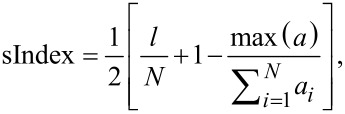


where *a* is the set of all electroconductive channel sizes in the image. *N* is the total number of electroconductive channels, and *l* is the number of electroconductive channels the area of which is less than the threshold of five pixels (ca. 980 nm). The threshold was selected because most of the channels are in the range of up to five pixels, and those that are not can be thought of as agglomerated. max(*a*) is the biggest electroconductive channel in the image.

The overall sample characterization index is acquired by taking the average of the previous two:

[3]
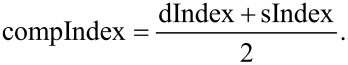


### Sensing properties

**Figure 2:**
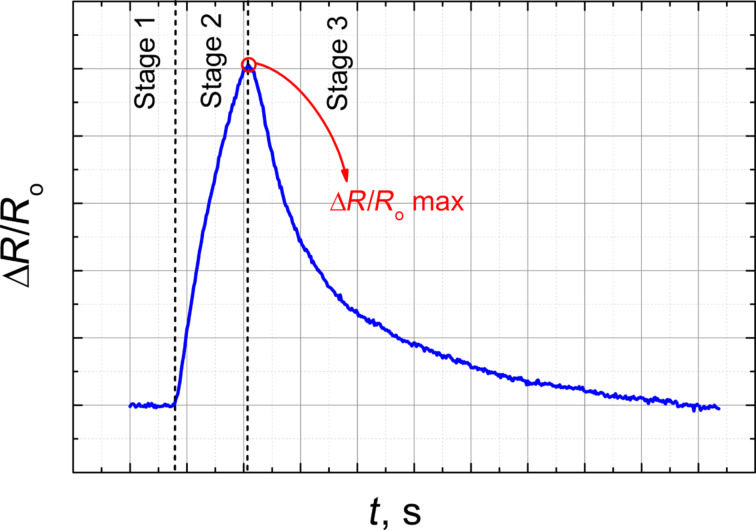
Expected relative change of the electrical resistance as a function of the time at different stages of the measurement. With the sensing effect we mean the peak of Δ*R*/*R*_0_ max in the second stage.

The sensing mechanism of the material is based on the diffusion of VOC molecules in the EVA–CB layer. Vapour molecules adsorb on the EVA–CB surface and then diffuse into the composite material. The polymer matrix swells and the distance between the CB nanoparticle aggregates increases. The magnitude of the tunnelling currents between the CB aggregates decreases with an increased distance and therefore the electrical resistance of the composite increases [21]. In this study, “sensing effect” means the effect illustrated in Figure 2 and described below.

**Figure 3:**
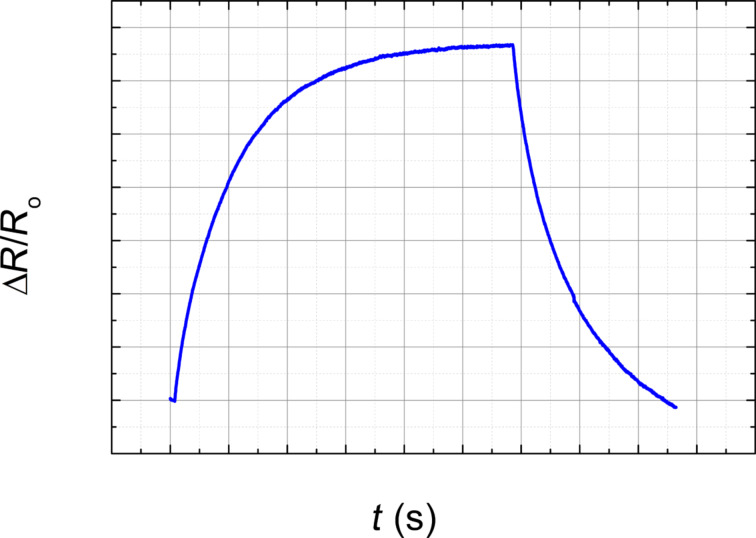
Expected relative electrical resistance change as a function of time in which the curve approaches saturation.

Stage 1 is the determination of the initial resistance of the sample as a function of the time in clear air or synthetic air flow (200 sccm). Stage 2 is the exposure: The change of the electrical resistance is measured when VOC vapours are applied in a certain concentration for a certain amount of time. Stage 3 is the relaxation: The electrical resistance gradually decreases to the initial value. During the three stages the relative change of the electrical resistance varies as shown in Figure 2. The highest peak of this curve is marked as Δ*R*/*R*_0_ max. This is the maximal relative change of the electrical resistance at the end of the exposure time, for example, at 60 s. By changing the exposure time, the value of Δ*R*/*R*_0_ max and the relaxation time will change. If the exposure time is long enough then the curve approaches saturation (Figure 3).

## Results and Discussion

### Composite characterization

**Figure 4:**
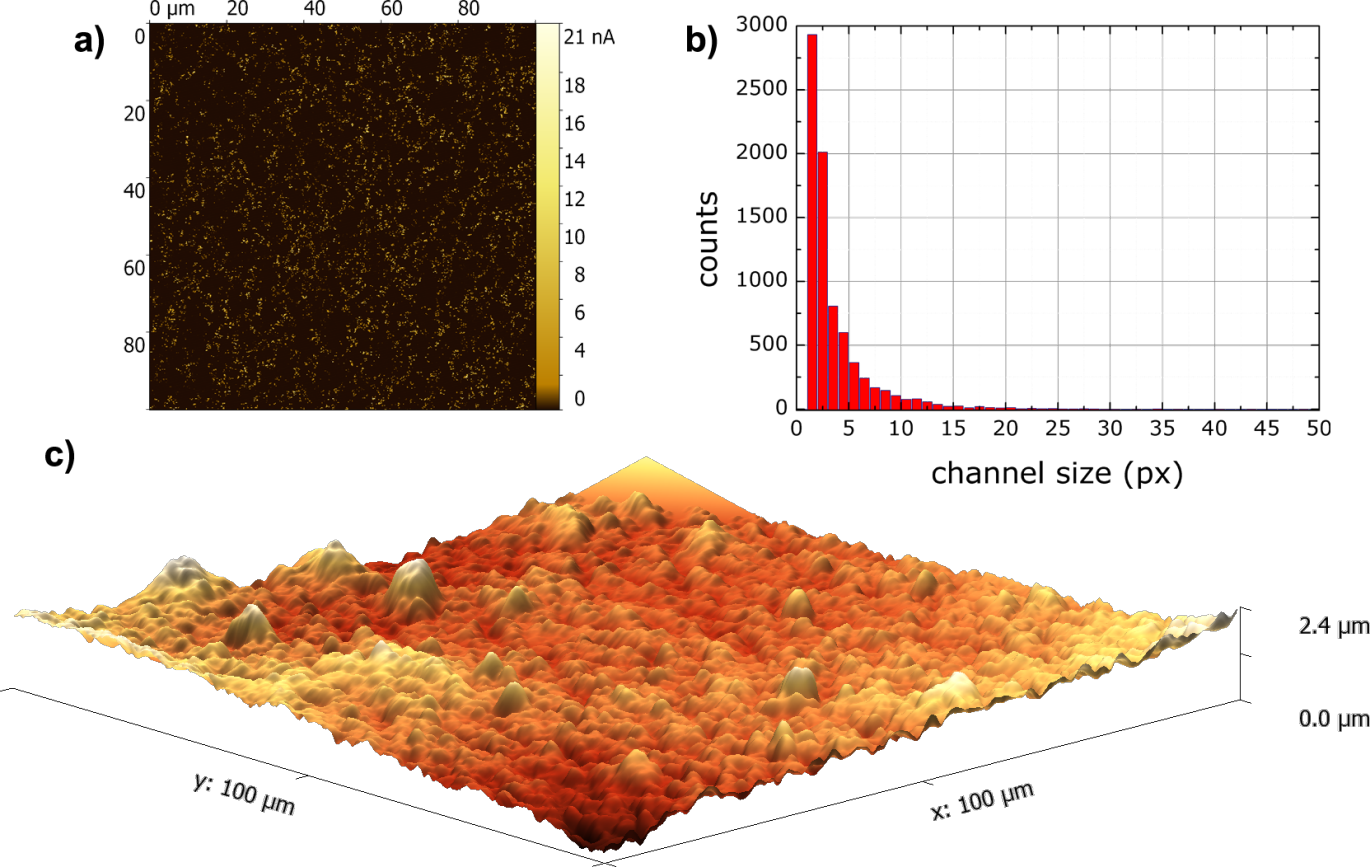
(a) Electroconductive map of CB channels of EVA–CB (7.75 phr CB) and (b) channel size distribution histogram; (c) surface AFM image.

Figure 4a shows a typical AFM electroconductive-channel image of an EVA–CB composite with 7.75 phr CB. The channels are distributed quite evenly throughout the whole image with little agglomeration. A channel is visible if the electrical current that flows through the sample is greater than 170 pA. The channels vary in current strength from 170 pA to 20.6 nA. Figure 4b shows the channel size distribution histogram. The channels vary in size from 1 to 48 pixels and one pixel is approximately 196 nm. The dIndex for this image is 0.917, the sIndex is 0.907, and the resulting compIndex is 0.912. dIndex characterizes the overall distribution of the electroconductive channels and is smaller if channels are more abundant in one particular segment [24]. In this image the channels are distributed very evenly throughout the whole image so the index is close to one. sIndex characterizes the channel-size distribution and decreases if the cross section of the electroconductive channels is larger than 5 pixels (ca. 980 nm). The average channel cross section is three pixels (ca. 590 nm) and 95% of channels are in the range of up to 10 pixels, so the index is close to one.

### Sensing measurements

**Figure 5:**
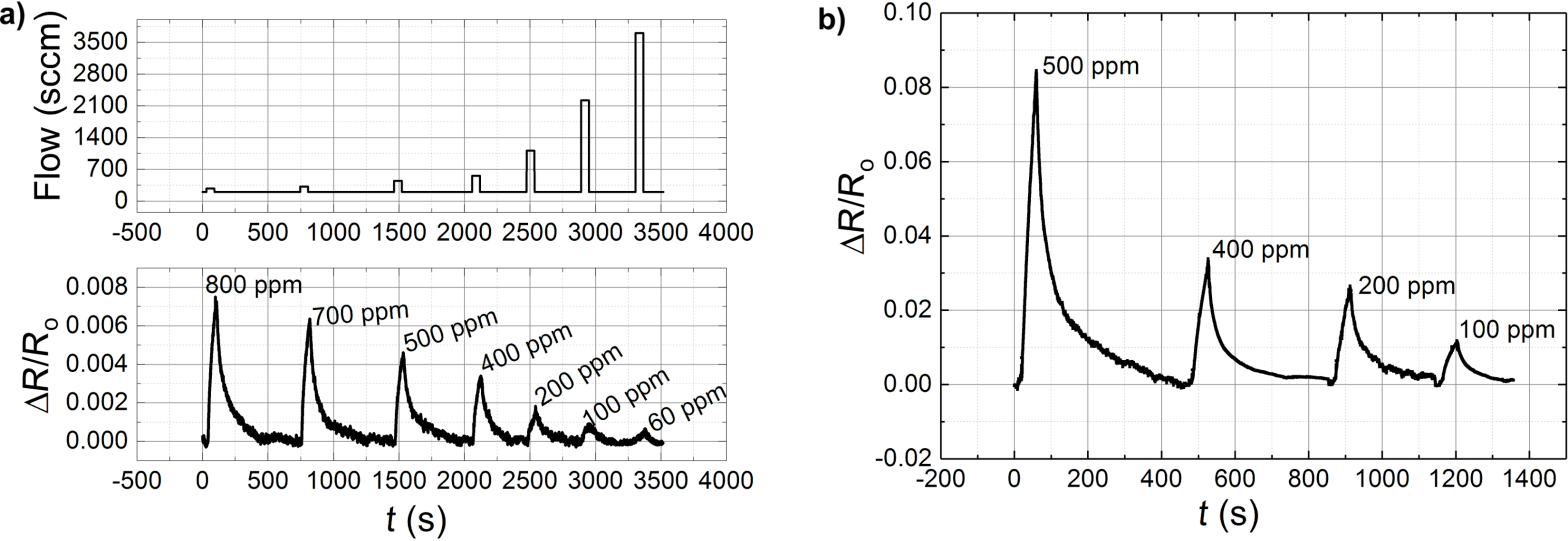
Relative change of the electrical resistance of EVA–CB (7.75 phr CB) as a function of time in a) benzene and b) ethylbenzene vapours (60 s exposure) for various vapour concentrations. Sample thickness is 50 µm.

**Figure 6:**
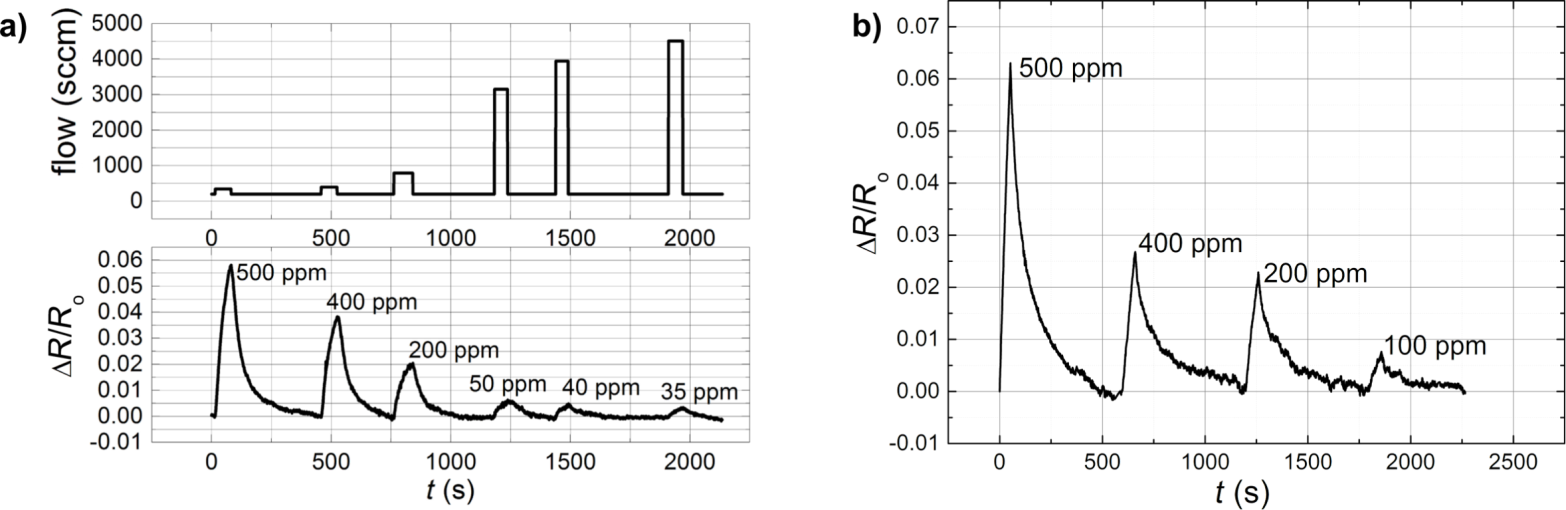
Relative change of the electrical resistance of EVA–CB (7.75 phr CB) as a function of time in a) toluene and b) *m*-xylene vapours (60 s exposure) for various vapour concentrations. Sample thickness is 50 µm.

The nanostructured composite samples (EVA-CB) were exposed to BTEX vapours in order to determine the EVA-CB response to these vapours. We present vapour sensing measurements of benzene (Figure 5a) and ethylbenzene (Figure 5b) and toluene (Figure 6a) and *m*-xylene (Figure 6b) in various concentrations. In all four situations ∆*R*/*R*_0_ max increases with increased vapour concentration just like described previously. The change of electrical resistance at different flow rates, without any analyte was also measured. It turned out to be minimal with an opposite tendency, i.e. the value of electrical resistance drops with increasing flow rate. However, the change was within the amplitude of the sensor signal noise. In case of benzene and toluene the sensing measurements were made using the Kin-Tek FlexStream automated permeation tube system. As expected the measured effect decreases with increasing Kin-Tek carrier gas flow (Figure 5a) because the faster gas flow does not allow the molecules to adsorb.

In Figure 5 we can see that at 500 ppm, the value of ∆*R*/*R*_0_ max for ethylbenzene is over 0.08 while for benzene it is over 0.004. Usually a VOC with smaller molecules shows higher values in the sensing measurement, but here it is clear that ethylbenzene (the solvent with a bigger molecule) shows better sensing-measurement results than benzene. It could be that ethylbenzene molecules are bigger but still small enough to diffuse into the polymer matrix inducing a swelling of the composite and an increase of the distance between CB nanoparticle aggregates. In case of benzene more molecules diffuse into the polymer matrix, but they increase the distance between the CB nanoparticles aggregates to a lower degree than in case of ethylbenzene. Figure 6 shows similar ∆*R*/*R*_0_ max values for toluene and *m*-xylene at a concentration of 500 ppm. This is due the similar molecular structures and sizes for toluene and *m*-xylene.

**Figure 7:**
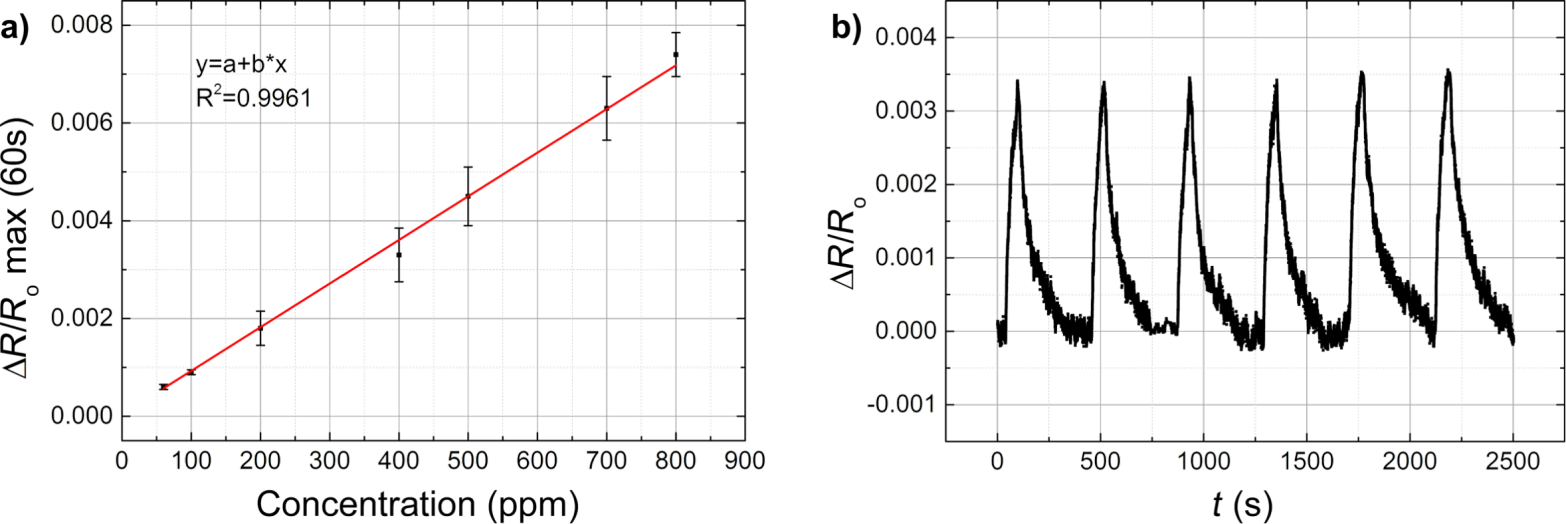
a) Δ*R*/*R*_0_ max values of EVA–CB (7.75 phr CB) after 60 s exposure to various concentrations of benzene vapours. The error bars show the scattering for parallel sample measurements; b) Δ*R*/*R*_0_ max values of one sample of EVA–CB (7.75 phr CB) repeatedly exposed to benzene vapours (400 ppm, 60 s). The sample thickness is 50 µm.

Figure 7 shows the reproducibility of the measurements with one EVA–CB sample (Figure 7b) and for several parallel samples (Figure 7a). In case of one sample the reproducibility is adequate, which indicates that EVA–CB could be used several times in a row. In case of several parallel samples (Figure 7 a) the scattering between the samples is higher than the scattering in case of one sample, but the results are still relatively close. The scattering between parallel samples leads to difficulties with a precise concentration determination. However, Figure 7a shows that the response increases linearly with an increasing concentration.

**Figure 8:**
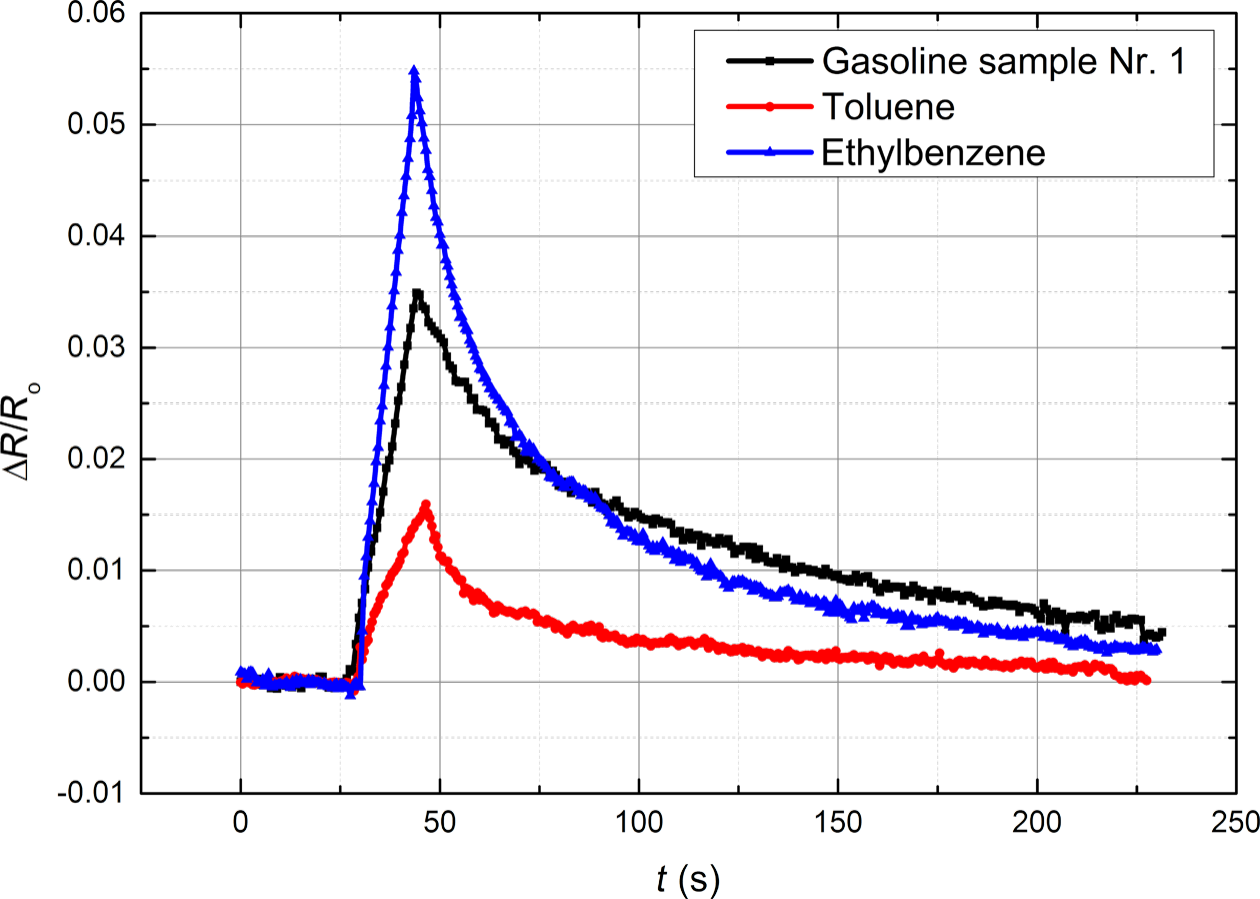
Relative change of the electrical resistance of EVA–CB (7.75 phr CB) as a function of time in different vapours after exposure for 15 s (0.0037 mL/L). The sample thickness is 50 µm.

Figure 8 shows the sensing measurements of benzene derivatives (toluene and ethylbenzene) in comparison with gasoline vapours. The effect for ethylbenzene vapours is the largest and for toluene it is the smallest. The response to gasoline vapours is between the former two. This could be explained with the structure of the molecules. Ethylbenzene is bigger than toluene and as mentioned previously ethylbenzene molecules induce a swelling of the polymer matrix more effectively than VOCs with smaller molecule size such as toluene. Ethylbenzene and toluene are pure compounds, but the contents of gasoline may vary based on its production including many highly volatile substances that can affect the response.

## Conclusion

This study demonstrates that EVA–CB (7.75 phr CB) can be used to detect gasoline and some of its contents such as BTEX vapours. EVA–CB showed a clear reproducible response to BTEX as well as gasoline vapours, and the response increases linearly with increasing vapour concentrations.

EVA–CB can be employed to detect different BTEX vapours, but there is a scattering between the sensing measurements of parallel samples. This makes a precise concentration determination more difficult. Therefore, there is a need to improve the sensor in order to decrease the scattering and optimize the detection range. The addressed concentration ranges in this study were for benzene 60 to 800 ppm, for ethylbenzene 100 to 500 ppm, for toluene 35 to 500 ppm, and for *m*-xylene 100 to 500 ppm.

This study showed that EVA–CB is a promising composite material that can detect gasoline and BTEX vapours and could be applied in future for fuel quality control.
